# The sinonasal communication in the horse: examinations using computerized three-dimensional reformatted renderings of computed-tomography datasets

**DOI:** 10.1186/1746-6148-10-72

**Published:** 2014-03-19

**Authors:** Markus Brinkschulte, Astrid Bienert-Zeit, Matthias Lüpke, Maren Hellige, Bernhard Ohnesorge, Carsten Staszyk

**Affiliations:** 1 Clinic for Horses, University of Veterinary Medicine Hannover, Foundation, Bünteweg 9, Hannover D-30559, Germany; 2 Institute for General Radiology and Medical Physics, University of Veterinary Medicine Hannover, Foundation, Bischofsholer Damm 15, Hannover D-30173, Germany; 3 Department of Veterinary-Anatomy, -Histology, and -Embryology, Faculty of Veterinary Medicine, Justus-Liebig-University Giessen, Frankfurter Str. 98, Giessen D-35392, Germany

**Keywords:** Horse, Computed tomography, Nasomaxillary aperture, Apertura nasomaxillaris, Upper airway, Sinonasal channel system, Paranasal sinuses

## Abstract

**Background:**

Sinusitis is a common disease in the horse. In human medicine it is described, that obstruction of the sinonasal communication plays a major role in the development of sinusitis. To get spatial sense of the equine specific communication ways between the nasal cavity and the paranasal sinuses, heads of 19 horses, aged 2 to 26 years, were analyzed using three-dimensional (3D) reformatted renderings of CT-datasets. Three-dimensional models were generated following manual and semi-automated segmentation. Before segmentation, the two-dimensional (2D) CT-images were verified against corresponding frozen sections of cadaveric heads.

**Results:**

Three-dimensional analysis of the paranasal sinuses showed the bilateral existence of seven sinus compartments: rostral maxillary sinus, ventral conchal sinus, caudal maxillary sinus, dorsal conchal sinus, frontal sinus, sphenopalatine sinus and middle conchal sinus. The maxillary septum divides these seven compartments into two sinus systems: a rostral paranasal sinus system composed of the rostral maxillary sinus and the ventral conchal sinus and a caudal paranasal sinus system which comprises all other sinuses. The generated 3D models revealed a typically configuration of the sinonasal communication ways. The sinonasal communication started within the middle nasal meatus at the nasomaxillary aperture (Apertura nasomaxillaris), which opens in a common sinonasal channel (*Canalis sinunasalis communis*). This common sinonasal channel ramifies into a rostral sinonasal channel (*Canalis sinunasalis rostralis*) and a caudo-lateral sinonasal channel (*Canalis sinunasalis caudalis*). The rostral sinonasal channel ventilated the rostral paranasal sinus system, the caudo-lateral sinonasal channel opened into the caudal paranasal sinus system. The rostral sinonasal channel was connected to the rostral paranasal sinuses in various ways. Whereas, the caudal channel showed less anatomical variations and was in all cases connected to the caudal maxillary sinus. Volumetric measurements of the sinonasal channels showed no statistically significant differences (*P* <*0.05*) between the right and left side of the head.

**Conclusions:**

Under physiologic conditions both paranasal sinus systems are connected to the nasal cavity by equine specific sinonasal channels. To resolve sinus disease it is aimed to maintain or even reconstruct the normal anatomy of the sinonasal communication by surgical intervention. Therefore, the presented 3D analyses may provide a useful basis.

## Background

Diseases of the equine paranasal sinuses are of special interest in equine medicine, since sinusitis is a common and widespread disease [1]. The detailed anatomy of the equine paranasal sinuses and the nasal cavity is and has been extremely challenging. However, detailed knowledge of these structures is essential for successful diagnostic and surgical intervention [2-4]. In human medicine, drainage is the most important factor for establishing physiologic conditions of the paranasal sinuses [5]. Furthermore, obstructions of the sinonasal pathways are suspected to be a primary factor in developing sinusitis in man [2].

The development of the equine paranasal sinuses starts during organogenesis. At this time the mucosa of the middle nasal meatus protrudes into the diploe of the maxillary bone; through which a two-parted anlage of the maxillary sinuses (separating into the rostral and caudal maxillary sinus) is developed [6]. This embryological concept explains the anatomical finding that all equine paranasal sinuses are connected to the middle nasal meatus via a nasomaxillary aperture (Apertura nasomaxillaris) [7,8]. The nasomaxillary aperture is described as a common entrance into the rostral and caudal maxillary sinus [6,8-12].

The rostral maxillary sinus (Sinus maxillaris rostralis, SMR) is connected to the ventral conchal sinus (Sinus conchae ventralis, SCV) establishing the rostral paranasal sinus system. The caudal maxillary sinus (Sinus maxillaris caudalis, SMC) is the central compartment of the caudal paranasal sinus system and opens into the dorsal conchal sinus (Sinus conchae dorsalis, SCD), the middle conchal sinus (Sinus conchae mediae, SCM), the frontal sinus (Sinus frontalis, SF) and the sphenopalatine sinus (Sinus sphenopalatinus, SSP). Because of continuous communication between the frontal sinus and dorsal conchal sinus a compound term for both compartments is conchofrontal sinus (Sinus conchofrontalis). But for superior visualisations in the current study both compartments are separated from each other and visualized with two different colours. Remarkably, the rostral and caudal paranasal sinus systems are completely separated by the maxillary septum [13]. Thus, the airways between the nasomaxillary aperture and the two sinus systems should be divided into at least two separated branches, one ventilating the rostral paranasal sinus system and another ventilating the caudal paranasal sinus system. However, the courses and dimensions of the connecting airways between the nasomaxillary aperture and the maxillary sinuses are not fully understood so far.

Such detailed knowledge and a spatial understanding of the communication ways between the nasal cavity and paranasal sinuses is essential for establishing new transnasal diagnostic and surgical procedures that maintain or reconstruct normal anatomy of diseased sinonasal pathways.

Therefore the aim of this study was to examine the spatial configuration and the volumetric expansion of the sinonasal communication in non-diseased horses. Further, anatomical terms are suggested to establish a precise and self-explanatory nomenclature for the complex anatomy of the equine sinonasal pathways.

## Methods

Nineteen equine cadaveric heads of various breeds (13 Warmbloods, 4 Thoroughbreds, 2 others; 8 mares, 8 geldings and 3 stallions) were used to acquire CT-datasets. The age of the examined horses ranged from 2 to 26 years (mean ± SD, 12.6 ± 8.3 y.). The horses were humanly euthanized for reasons unrelated to the head. None of the horses had a known history or clinical signs of paranasal sinus disease. Ethical approval for this study was obtained from the ethics committee within the University of Veterinary Medicine, Hannover, Germany. Post mortem, the heads were harvested in the atlanto-occipital joint, cooled (4°C) and CT-datasets were acquired within 24 hours (mean ± SD, min - max; 8:29 ± 6:30 h:mm, 1:46–21:11 h:mm) after euthanasia. Image acquisition was performed at the Clinic for Horses of the University of Veterinary Medicine Hannover, Foundation, with a multislice scanner (Brilliance^TM^ CT - Big Bore Oncology Scanner, Philips Medical Systems, Best, The Netherlands). The skulls were positioned standing on the mandible. Transverse images of the whole head were acquired in an axial scan-mode using the following parameters: 140 kV, 500 mAs, 1.5 mm slice thickness. One soft tissue series using a low frequency filter (512 × 512 image matrix) and one series for bony details using an edge enhancing filter (1024 × 1024 image matrix) were acquired. By the use of multiplanar reconstructions (MPR) different series were generated. Two transversal series (soft tissue: WL 50 Hounsfield Units (HU), WW 500 HU; bony detail: WL 300 HU, WW 2800 HU) oriented perpendicular to the hard palate and one coronal series (bony detail: WL 300 HU, WW 2800 HU) oriented parallel to the hard palate were reformatted. The 2D slices were viewed to obtain normal morphology and existence of pathological changes. Furthermore, positioning of the nasomaxillary aperture in relation to the teeth was noticed.

**Figure 1:**
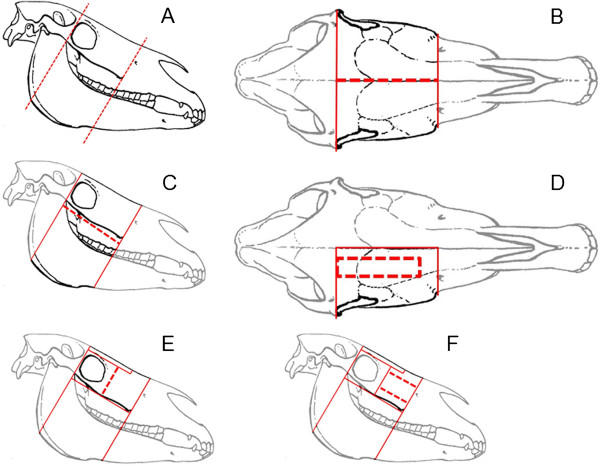
**Illustration of the dissection of cadaver heads.** The broken red lines show the actual cut, whereas the continuous red lines show the intersections: two transversal cuts, one rostral of the Crista facialis and another caudal to the orbital cavity **(A)**, a median sagittal cut **(B)**, a horizontal cut parallel to the Crista facialis **(C)**, a frontonasal bone flap **(D)**, removal of the orbital cavity **(E)** and a maxillary bone flap **(F)** were done.

After CT-image acquisition the heads were frozen and dissected by the use of a band saw (Kolbe Typ K420, Kolbe, Elchingen, Germany) and by use of an oscillating saw (Oscillant Typ G-6100-05, Aesculap, Tuttlingen, Germany) (Figure 1A, B). Horizontal cuts, frontonasal and maxillary bone flaps were performed to visualize the entrance of the sinonasal channel system into the paranasal sinuses (Figure 1C, D, E, F). To access the nasomaxillary aperture (Apertura nasomaxillaris) within the middle nasal meatus the nasal cavity was exposed by use of a diamond blade saw (Micro MBS 240/E, Proxxon, Föhren, Germany). The final specimens consisted of parts of the maxillary bone, maxillary septum, lamellae of the dorsal and ventral nasal concha and parts of the maxillary dentition. After examination the specimens were stored in Peter’s solution at 4°C.

The program Amira 5.3.3® (VSG, Mérignac Cedex, France) was used for segmentation of CT-datasets of the paranasal sinuses and sinonasal channels. The reconstructed transversal dataset (bony details, transversal orientated parallel to the hard palate) was uploaded and the sinonasal communication ways were reconstructed by use of manual segmentation slice by slice starting rostral. In order to generate consistent and reliable 3D models of 38 demi-heads distinctive anatomical landmarks were defined and used for segmentation in all 19 heads.

**Figure 2:**
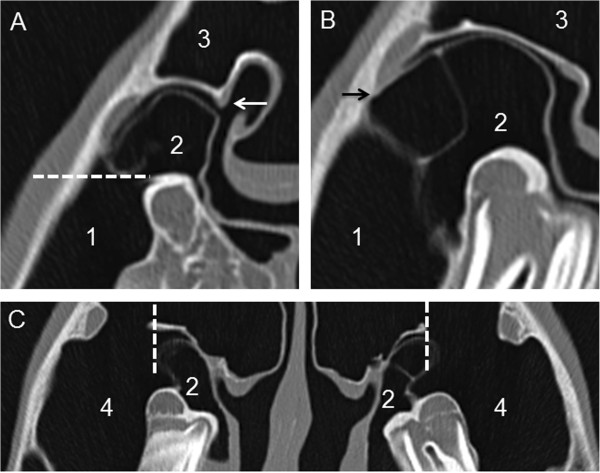
**Illustration of limits for segmentation of the sinonasal channel in transversal CT**-**slices.** In a CT-slice at the level of 109 **(A)**, the medial limitation for segmentation was a ‘hook’ protruding from the spiral lamella of the dorsal concha into the middle meatus (white arrow). The broken line shows the intrasinuidal limit for segmentation of the rostral sinonasal channel. In the CT-slice at the rostral level of 110 **(B)**, the fusion of the spiral lamella of the ventral concha with the maxillary bone can be seen (black arrow). At the caudal level of 110/210 **(C)**, the broken line displays the intrasinuidal limit for segmentation of the caudal sinonasal channel. Please note the differences of the ‘bulla’-like protrusion of the SCV (*Bulla septi sinuum maxillarium*) between left and right side. Numbered structures: (1) SMR, (2) SCV, (3) SF, (4) SMC.

The first slice for segmentation was defined to be the most rostral one, in which the sinonasal channel is in continuous communication with the paranasal sinuses (SMR or SCV). The landmark for the medial beginning of manual segmentation in the middle meatus was a typically seen bony ‘hook’ orienting dorsally from the lamella of the dorsal conchal sinus (Figure 2A). The lateral ending for manual segmentation of the communication ways was defined as: 1. horizontal line, drawn between the most ventral point of the spiral lamella of the ventral conchal sinus to the maxillary bone (Figure 2A), 2. limited by the fusion of the spiral lamella of the ventral concha with the maxillary bone (Figure 2B), 3. vertical line beginning ventrally at the maxillary septum, ending dorsally at the lamella which forms the floor of the dorsal conchal sinus and the frontomaxillary aperture (Figure 2C).

In a second step demarcation lines were manually drawn slice by slice to separate the paranasal sinus compartments from each other and from the nasal cavity. Semi-automated segmentation of the sinus cavities was performed inside the tissue lining of the sinuses by using a masking from -1100 to -600 HU. A seed point for a 2D growing region algorithm was set slice by slice in every compartment and the resulting segmented area was added to the contributing sinus. After semi-automated segmentation a manual correction of artefacts such as holes and fluid fillings was performed for each slice.

For surface calculation an unconstrained smoothing algorithm was applied to the reconstructed paranasal sinuses. No smoothing algorithm was applied to models of the sinonasal pathways.

After segmentation volumetric measurements of the generated 3D models of the communication ways were performed using Amira 5.3.3® (VSG, Mérignac Cedex, France).

### Statistical analyses

For data collection a spread sheet (Excel® 2010, Microsoft® Corporation Redmond, Washington, USA) and for further statistical analyses a separate software was used (Prism®, GraphPad Software, La Jolla, USA).

Descriptive statistics were performed. To determine the normality of distribution, a Kolmogorov-Smirnov test was assessed. A paired t-test was used to analyse if there were significant differences in the volume of the sinonasal channels between the left and the right side. The level of significance was set at *P* < *0.05*.

## Results

In all of the 19 analysed datasets the communication ways between the nasal cavity and the paranasal sinuses were seen with a typically mode of ramification.

**Figure 3:**
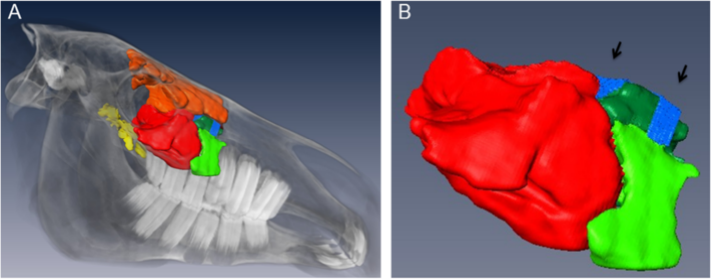
**3D models of the right side of a 3**-**year old Warmblood mare (lateral view).** The segmentation results of paranasal sinuses and sinonasal channels and the volume rendering of the bony structures of the head are shown **(A)**. The enlargement **(B)** of Figure 3-A visualizes the entrance of the sinonasal channels into the paranasal sinuses. Coloured structures: bright green: SMR, dark green: SCV, red: SMC, dark red: SCD, orange: SF, yellow: SSP; blue: sinonasal channel system.

As expected, seven sinus compartments were identified for both sides in the 2D datasets (Figure 3), which were divided in two totally separated sinus systems [13].

In 12 (63%) of the 19 datasets some of the sinus compartments contained some gravity dependent fluids. Macroscopic preparation verified these findings as serosanguineous fluids. In one case (26-year old Warmblood stallion) a homogeneous structure (25 HU; dimensions latero-lateral × dorso-ventral × rostro-caudal, 54.7 × 53.9 × 60.0 mm) was seen in the right SMC. This structure could be identified during macroscopic preparation as a fluid filled cyst, which was at the bottom of the SMC and without contact to the sinonasal channel system.

The nasomaxillary aperture was visible in the middle meatus in every transversal CT-dataset and on macroscopic preparation. In relation to the alveoli of the cheek teeth, the nasomaxillary aperture was located at the level of the first (109/209) and second (110/210) molar in horses younger than 15 years. In horses older than 15 years the nasomaxillary aperture was located at the level of the second (110/210) and third (111/211) molar.

Lateral to the slit-shaped nasomaxillary aperture a system of channels which were in communication with the paranasal sinuses were observed.

**Figure 4:**
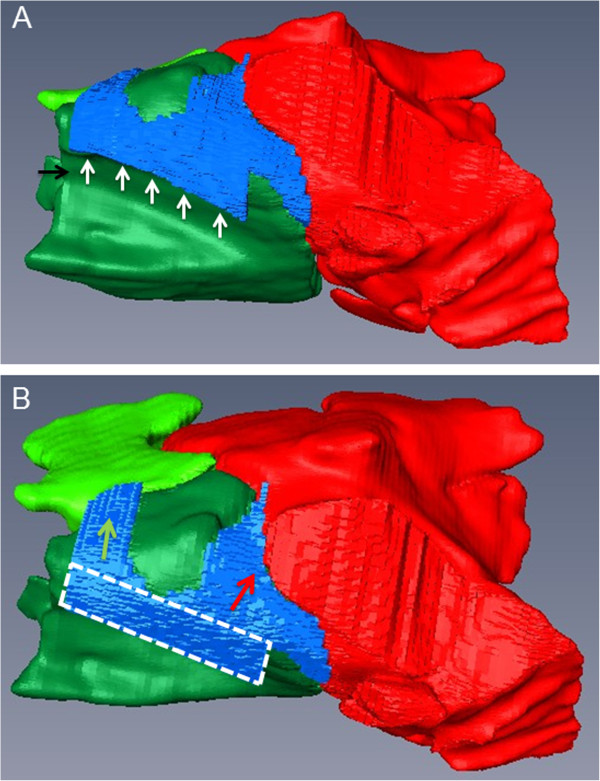
**3D models of the sinonasal channels and the SMR**, **SCV and SMC of a 3**-**year old Warmblood mare.** In a dorso-medial view of the right side **(A)**, the middle nasal meatus (black arrow), the nasomaxillary aperture (white arrows) and the sinonasal channel system (blue) are visible. The dorsal view gives an overview of the different parts of the sinonasal channels **(B)**: The broken lines border the common sinonasal channel (*Canalis sinunasalis communis*). The green arrow lies within the rostral sinonasal channel (*Canalis sinunasalis rostralis*), whereas the red arrow lies within the caudal sinonasal channel (*Canalis sinunasalis caudalis*). Coloured structures: bright green: SMR, dark green: SCV, red: SMC, blue: sinonasal channel system.

The generated 3D models of these communication ways showed a typically configuration which can be subdivided into three parts (Figure 4). The first part was a common sinonasal channel (*Canalis sinunasalis communis*^a^), medial starting at the nasomaxillary aperture in the middle meatus, dorsally limited by the spiral lamella of the dorsal concha and ventrally limited by the spiral lamella of the ventral concha. The caudal limitation was built by the fusion of the before mentioned lamellae. This part of the sinonasal channel was dorso-laterally oriented. In its further extent, the common sinonasal channel was divided into two distinct channels. This separation was caused by fusion of the ventral spiral lamella with the maxillary bone and resulted in a rostral channel, orienting laterally, and a caudal channel, orienting caudo-laterally. The rostral sinonasal channel (*Canalis sinunasalis rostralis*^*a*^) was connected to the rostral sinus system. Coming from dorso-medial it turned ventrally; where it got access to the paranasal sinus compartments of the rostral sinus system.

**Figure 5:**
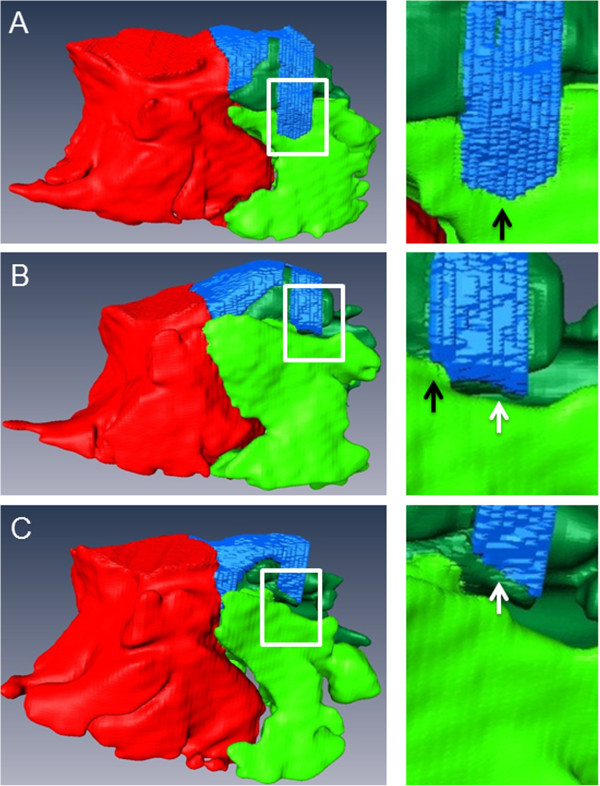
**3D models of the sinonasal channels and SMR**, **SCV and SMC of three different horses.** All pictures show the right side in a lateral view. In a 8-year old Warmblood gelding **(A)**, the rostral sinonasal channel was in single communication with the SMR. In another horse **(B)**, a 7-year old Thoroughbred gelding, the rostral sinonasal channel was in communication with the SMR and the SCV. Whereas, in a 26-year old Warmblood stallion **(C)**, the rostral sinonasal channel was in single communication with the SCV. Coloured structures: bright green: SMR, dark green: SCV, red: SMC, blue: sinonasal channel system.

There was some variation in the entrance of this channel in the rostral sinus system (Figure 5). In most head sides (20 of 38; 52.6%) the rostral sinonasal channel was solely connected to the SMR. In 16 of 38 cases (42.1%) it was in communication with the SMR as well as with the SCV. In the remaining two cases (2 of 38; 5%) the rostral sinonasal channel was in sole connection with the SCV. Comparing the left and the right side, in eight horses (8 of 19; 42.1%) on both sides this channel was solely connected with the SMR. In six horses (6 of 19; 31.6%) the channel was bilaterally connected to both compartments (SMR and SCV). Furthermore, in four horses (4 of 19; 21.0%) this connection to both compartments could be seen on one side, whereas the other side was solely connected to the SMR. In one horse for both sides, the rostral sinonasal channel was in single communication with the SCV (1 of 19; 5.3%).

In contrast, the caudal sinonasal channel (*Canalis sinunasalis caudalis*^*a*^) depicted less anatomical variations and was in all cases directly connected to the SMC (38 of 38). Orienting from the common sinonasal channel it turned caudo-laterally. It was dorsally limited by the spiral lamella of the dorsal concha, which built the ventral roof of the SCD. The ventral limitation was built by the dorsal part of the maxillary septum, which was the caudally oriented, ‘bulla-like’ protrusion of the SCV into the SMC (*Bulla septi sinuum maxillarium*^*a*^). There was some variation in the extent of the caudal sinonasal channel, due to the size of the ‘bulla-like’ protrusion of the SCV. In relation to this caudal protrusion in 24 of 38 head sides (63%) the caudal sinonasal channel was in contact to rostro-medial parts of the frontomaxillary aperture. This could be seen for both sides in 13 of 19 horses (68%).

**Figure 6:**
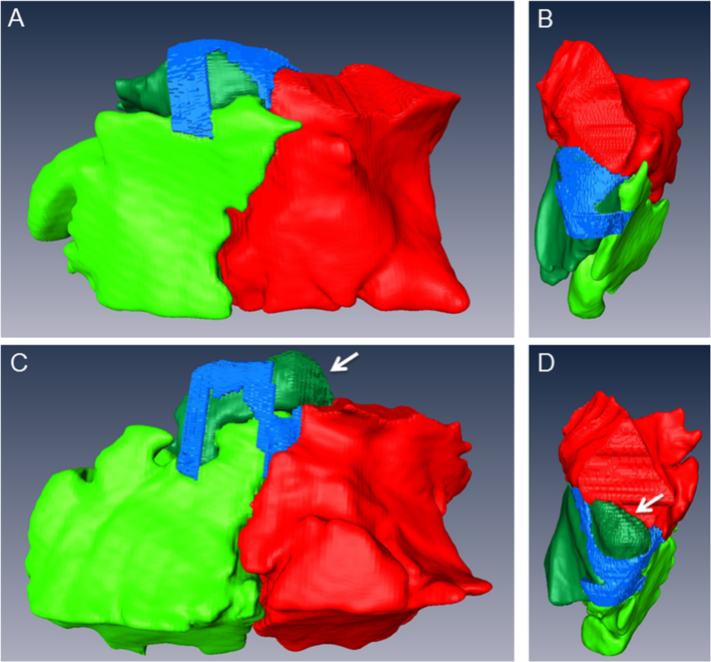
**3D models of the sinonasal channels and the SMR**, **SCV and SMC of two different horses.** The sinonasal channels (blue) and the SMR (bright green), SCV (dark green) and SMC (red) of the left side of a 20-year old Warmblood gelding **(A)**, **(B)** and a 20-year old Warmblood mare **(C)**, **(D)** are shown in a lateral view **(A)**, **(C)** and dorsal view **(B)**, **(D)**. Please note the large, caudo-dorsal oriented protrusion (*Bulla septi sinuum maxillarium*) of the SCV in the second horse **(D)**, which leads to a subdivision of the caudal sinonasal channel (*Canalis sinunasalis caudalis*).

In one horse (20 years old Warmblood mare) the caudal sinonasal channel was subdivided into two parts, because of a caudo-dorsal protrusion of the SCV (Figure 6).

The total volume of the sinonasal channel system was at median 2.44 ml for the left (min - max; 1.64 to 14.7 ml) and 2.38 ml for the right side (min - max; 1.12 - 11.67 ml). There was no significant difference in the volume of the sinonasal channel system comparing the left and right side (*P* = *0.197*). The difference between the minimum and maximum total volume of the channels, reveal the individual variability in the diameter of the channel system. In some cases in the CT-datasets, and during macroscopic preparation, the space between the limiting lamellae was nearly invisible. Inserting a flexible plastic probe into the pathways during macroscopic preparation without destroying the membranes revealed that the pathways were in continuous communication with the middle nasal meatus, except for those areas where the spiral lamella of the ventral concha fused with the maxillary bone.

## Discussion

The generated 3D models represent the equine sinonasal pathways, which connect the paranasal sinuses with the nasal cavity. The ability to display the sinonasal channels singly or in addition with the paranasal sinus compartments, the viewer is able to get a spatial impression of these complex anatomical structures. In literature, there are several studies published describing this structure by use of macroscopic preparation [7,9,11,14] or CT imaging [15]. In contrast to traditional casting methods where the communication ways were closed to fill the paranasal cavities with plastoid [7], 3D models of the sinonasal channels could be generated by use of virtual casting methods. To the authors knowledge this is the first time that manual segmentation was used to generate 3D models of the equine sinonasal channels and that volumetric measurements for the evaluation of contralateral differences were performed.

In literature, there are varying descriptions of the equine sinonasal pathways. Most authors agree, that the nasomaxillary aperture provides a common entrance into the maxillary sinuses [8,9,11,16]. The nasomaxillary aperture is a slit-like channel, dorsal and ventral limited by the lamellae of the dorsal and ventral conchal sinuses, positioned above the second (110/210) and third (111/211) molar [7,15,17]. In agreement with this, we found the entrance into the sinonasal channel as a slit-like opening, located in the middle meatus in all of the 38 examined head sides. The position of this entrance varied with age. The results of our study show, that in younger horses the opening was located more rostrally compared to descriptions in literature and compared to older horses (≥ 15 year old), where it was positioned above the second and third molar. This can be due to a so called age related rostral drift of the cheek teeth [12,18].

Respecting the nomenclature for the openings between the sinus compartments [8,19]: conchomaxillary aperture (Apertura conchomaxillaris), frontomaxillary aperture (Apertura frontomaxillaris), palatomaxillary aperture (Apertura palatomaxillaris), we redefined the opening into the sinonasal channel system, the nasomaxillary aperture (Apertura nasomaxillaris). This rostro-caudally and horizontal oriented, slit-like opening was limited by a typically seen ‘hook’ , which protruded from the lamella of the dorsal concha ventrally into the middle meatus. This was defined as the beginning of the sinonasal channel system. We concluded that from a critical point of view only this opening can be called nasomaxillary aperture (Apertura nasomaxillaris). The nasomaxillary aperture led into a common sinonasal channel and, in agreement with other authors [6,8,9,11,12], by this it formed a common entrance to the paranasal sinuses. In the following this common sinonasal channel was divided into two separate channels, as described by others [7,9,11], which were in communication with a rostral and caudal sinus system. Our results also show that the caudal sinonasal channel varied less in its path into the SMC compared to the rostral sinonasal channel. As described in literature [7,17], there is some variation in the existence of this caudal sinonasal channel in relation to the caudal or dorso-caudal protrusion (*Bulla septi sinuum maxillarium*^*a*^) of the SCV. This protrusion forms the caudo-dorsal part of the maxillary septum and furthermore, the ventral limitation of the caudal sinonasal channel. When there is a large caudal protrusion of the SCV into the SMC, the intrasinuidal exit of the caudal sinonasal channel can be found directly rostral to the frontomaxillary aperture. In the current study in one case the dorsal protrusion of the SCV led to a separation of this caudal sinonasal channel into two parts.

In literature there are varying descriptions about the pathway leading into the rostral maxillary sinus. In the current study, in all cases a pathway between the nasal cavity and the rostral sinus system existed. In agreement with this, other authors [7,9,11] always found an entrance into the rostral sinus system. In the current study, this rostral sinonasal channel showed much variation in its communication with the two compartments of the rostral sinus system. In contrast, other authors describe, that the rostral pathway was always in communication with the SMR [7,9,11]. One author states [17], that there is a pathway into the SMR in cases, when the lamella of the ventral concha protrudes into the SMR. If this protrusion is absent, the lamella may be fused to the maxillary bone and the pathway is missing. We constantly found a fusion of the lamella of the ventral concha with the maxillary bone located between the rostral and caudal sinonasal channel. This separates both channels from each other, which is in agreement with other descriptions [11]. Others also analysed the communication ways between the nasal cavity and the paranasal sinuses by use of CT [15]. The nasomaxillary aperture was described as a connection between the SMC and the nasal cavity, whereas a connection between the SMR and the nasal cavity is not mentioned [15]. This can be due to the generated slice thickness. In the present study transversal slices with a thickness of 1.5 mm and 1024-image matrix were acquired, which resulted in high-resolution images. In contrast, Probst et al. (2005) examined transversal CT-datasets with a slice thickness of 5 mm and 512-image matrix [15].

From a clinical point of view, we state that for establishing physiologic conditions of the paranasal sinuses, the existence and functionality of these sinonasal channels is essential. In human medicine it is described that drainage of the paranasal sinuses depends on five factors: 1. the communication pathways, 2. the secretion, 3. the quality of the secrets, 4. the cilia activity and 5. the resorption [5]. In equine medicine some authors constitute that primary sinusitis leads to inflammation of the mucosa with following occlusion of the drainage pathways and accumulation of fluids in the paranasal sinuses [20]. Other authors describe, that the initial step in establishing paranasal sinus disease is the stagnation of mucociliary clearance, which can become chronically and lead to a mucosal hyperplasia. This hyperplasia contributes to the pathways occlusion [21]. In literature, mucosal hyperplasia in paranasal sinus disease up to 15 mm is described [22]. These authors concluded that the combination of mucosal swelling and lowered mucociliary clearance are predisposing for bacterial infections. Also the congenital missing of the natural openings is described to result in fluid accumulation within the paranasal sinuses and leading to a mucocele [23].

In human medicine functional endoscopic sinus surgery (FESS) is a minimal-invasive method to re-establish ventilation and drainage of the paranasal sinuses via the natural openings [24]–[26]. By re-establishing the natural pathways, there is no need for fenestration of the paranasal sinus at another area [25]. Moreover, in veterinary medicine balloon sinuplasty is described as a minimal-invasive treatment method for primary sinusitis [27]. After widening the caudal sinonasal channel, the flow-rate of fluids from the SMC into the middle nasal meatus was significantly higher than before, whereas dilatation of the rostral sinonasal channel was not possible. This reflects that detailed anatomical knowledge and a spatial sense of these structures are essential for diagnostical and surgical intervention. By viewing the 3D models of the sinonasal channel system and knowing the relations to other anatomical structures, the results of the balloon dilatation can be interpreted. The caudal sinonasal channel originates at the common sinonasal channel, turns caudolaterally and ends in the SMC. This channel is ventrally limited by the thin dorsal part of the maxillary septum, which is the caudal, ‘bulla-like’ protrusion of the ventral conchal sinus (*Bulla septi sinuum maxillarium*^*a*^). Depression of this part was visible in every horse after dilatation [27]. The entrance into the rostral sinus system is much more complicated, due to angulation of the rostral sinonasal channel and the elasticity of the ventral limitation of this channel. The ventral limitation is formed by the spiral lamella of the ventral concha. The fusion of this lamella with the maxillary bone resulted in returning of this lamella to its basic position after removing plastic probes from the rostral sinonasal channel, which was visible during macroscopic preparation. This lamella seemed to be fixed in position. Even if there is transnasal access to this part of the sinonasal channel system, which was rarely possible due to the 90° angulation [27], balloon sinuplasty of this part of the sinonasal channel system may be of limited value. If instruments are optimized for transnasal assessment of the rostral sinonasal channel, the insertion of drug eluting biodegradable stents as described in human medicine [26] could be a novel, minimal-invasive, successful technique.

In one study the procedure of equine paranasal sinusography is described [28]. By placing 130 ml contrast medium into the conchofrontal sinus, small amount of contrast agent was visible in the SMR. Fluids from the caudal sinus system can flow through the caudal sinonasal channel into the common sinonasal channel. It is the authors’ assumption that from here fluids can drain either via the nasomaxillary aperture into the nasal cavity or via the rostral sinonasal channel into the rostral sinus system. This fluid flow would be a gravitational dependent flow in relation to head position. Clinicians should be aware of this fluid flow, because pathological conditions of the caudal sinus system can be due to fluid fillings of the rostral paranasal sinus system without a primary pathological cause within the rostral sinus compartments.

## Conclusions

The natural pathways of the equine paranasal sinuses and the nasal cavity are a complex channel system, medially starting in the middle nasal meatus. The nasomaxillary aperture is the entrance into a sinonasal channel system, which is initially established as a common sinonasal channel (*Canalis sinunasalis communis*). In the following the channel is divided into two separate channels: 1. rostral sinonasal channel (*Canalis sinunasalis rostralis*) and 2. caudal sinonasal channel (*Canalis sinunasalis caudalis*). The rostral sinonasal channel is in communication with the compartments of the rostral sinus system: 1. in single communication with the SMR, 2. in communication with both compartments of the rostral sinus system (SMR and SCV), or 3. in single communication with the SCV. The caudal sinonasal channel is in communication with the SMC. Detailed anatomical knowledge is essential for successful diagnosis and surgical treatment. The generated 3D models give an excellent spatial impression of this anatomical structure. The suggested terms complete the existing nomenclature and contribute to a clear address of the respective part of the channel system, which is essential for diagnosis and further minimal-invasive treatment methods. Generated 3D models can be also used for educational purposes. Furthermore, these models allow virtual simulations to generate new transnasal, minimal-invasive surgical approaches and instruments.

### Endnote

^a^Terms written in italic are the authors’ suggestions for additional nomenclature. But up to now these terms are not listed in the Nomina Anatomica Veterinaria (2012).

## Abbreviations

3D: Three-dimensional; CT: Computed-tomography; SMR: Sinus maxillaris rostralis; SCV: Sinus conchae ventralis; SMC: Sinus maxillaris caudalis; SCD: Sinus conchae dorsalis; SF: Sinus frontalis; SSP: Sinus sphenopalatinus; SCM: Sinus conchae mediae; HU: Hounsfield units; WL: Window level; WW: Window width.

## Competing interests

None of the authors has any personal or financial relationships which could inappropriately influence or bias the content of this paper.

## Authors’ contributions

MB designed the study, optimized CT settings, acquired CT-data, performed segmentation and macroscopic preparations, analysed data, and wrote the manuscript. AB, CS and BO contributed to the study design, data analysis and interpretation and helped editing the manuscript. CS supervised macroscopic preparations. ML helped performing the segmentation. MH supported data acquisition and optimized CT settings. All authors read and approved the final manuscript.

## Authors’ information

Dr. Markus Brinkschulte and Dr. Astrid Bienert-Zeit are joint first authors.
